# Ileocecal knotting in a young man: A very rare and unique cause of intestinal obstruction

**DOI:** 10.1002/ccr3.3279

**Published:** 2020-08-30

**Authors:** Hailu Wondimu Gebresellassie

**Affiliations:** ^1^ Department of Surgery Addis Ababa University, College of Health Sciences, School of Medicine Addis Ababa Ethiopia

**Keywords:** intestinal obstruction, knotting, shock, short bowel syndrome

## Abstract

Ileocecal knitting should be considered as a differential diagnosis of small intestinal obstruction. Aggressive resuscitation and early surgery will be life‐saving. One should anticipate finding extensive bowel gangrene which requires aggressive resection.

## Graphical abstract

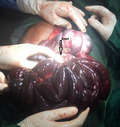

## 1 INTRODUCTION

The causes of bowel obstruction may be external to the bowel (extrinsic), within the wall of the bowel (intrinsic), or due to a luminal defect that prevents the passage of gastrointestinal contents. Most common causes of small bowel obstruction are adhesions, hernias, and small intestinal volvulus while large bowel obstruction is often caused by tumors and volvulus.^1^, ^2^

Advanced bowel obstruction leads to bowel dilation and retention of fluid within the lumen proximal to the obstruction, while bowel decompression occurs distal to the obstruction as luminal contents pass. If bowel dilation is excessive, or strangulation occurs, perfusion to the intestine can be compromised, leading to necrosis or perforation, complications that increase the mortality associated with small bowel obstruction.^1^

Intestinal knot syndromes are rare but life‐threatening cause of closed double‐loop obstruction. Various types of intestinal knot syndromes have been described such as ileosigmoid, ileoileal, appendico‐ileal, ileocecal, and knotting by meckel's diverticum. Ileosigmoid knotting is the commonest variety reported in the literatures. In this situation, devitalization of both segments occurs, and the prognosis is grave unless urgent surgical intervention is done.^3^, ^4^, ^5^, ^6^

Preoperative diagnosis of intestinal obstruction due to knotting is quite difficult if not impossible.

Ileocecal knotting occurs when a loop of ileum wraps around a mobile cecum and ascending colon. It commonly presents with acute small bowel obstruction and needs urgent resuscitation and surgical intervention.^7^ Occasionally, a subacute presentation which responds to conservative management has also been described. Surgical management often requires resection of gangrenous bowel and end to end anastomosis.^8^

In this paper, we present a 21‐year‐old male Ethiopian patient who presented in a critical condition with signs and symptoms of small bowel obstruction but found to have ileocecal knotting with mobile cecum and ascending colon.

## 2 CASE REPORT

A 21‐year‐old young Ethiopian man with a BMI of 24.6 kg/m^2^ presented with abdominal cramps, bilious vomiting, and abdominal distention of 48 hours duration to Zewditu Memorial Hospital, Addis Ababa, Ethiopia. He had no history of similar illness before, and there was no history of prior surgery. The pain was severe, centrally located with no radiation. There was no history of fever and bloody stool.

At presentation, he was acutely sick looking with feeble pulses and a blood pressure of 60/40 mm of mercury. Only 60 mL of urine was collected at initial catheterization. Abdomen was moderately distended with direct and rebound tenderness all over and absent bowel sounds. Rectum was empty on per rectal examination.

**FIGURE 1:**
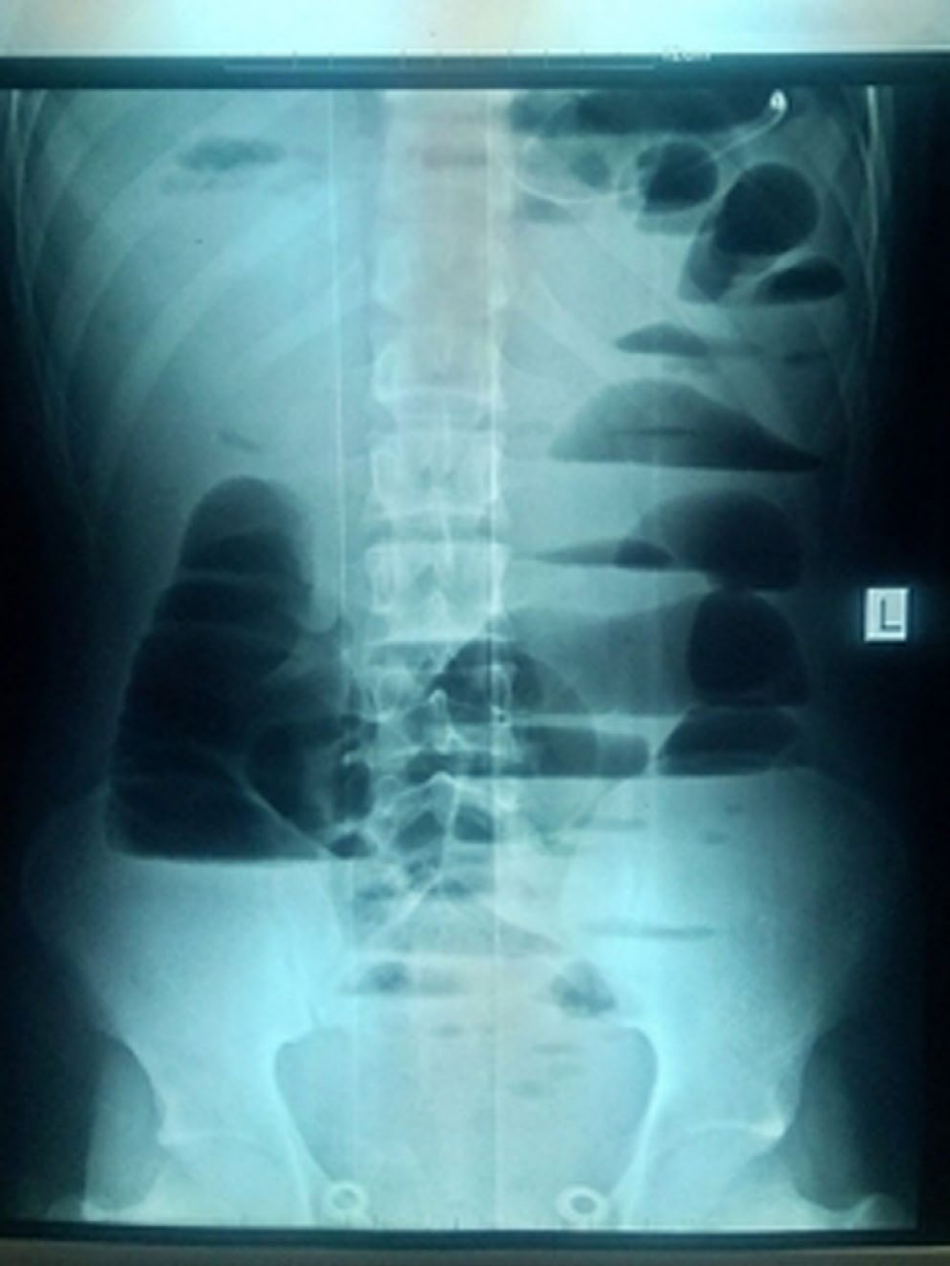
An erect plain film of the abdomen showing multiple air fluid and distended small bowel suggestive of small bowel obstruction

Two IV lines were secured with wide bore cannulas and aggressive resuscitation with 5 L of crystalloids started. His blood pressure improved to 100/60 mm of mercury and his pulse become well palpable and he produced adequate urine. Investigations revealed white cell count of 18 000 and hemoglobin of 15.49 g/dL. Erect abdominal X‐ray showed multiple air‐fluid level and distended bowel loops suggestive of small bowel obstruction (Figure 1).

Diagnosis of acute abdomen with gangrenous small bowel obstruction was made and urgent surgery decided.

He was then explored through a long midline incision. Exploratory laparotomy showed a very mobile cecum and ascending colon (not attached to posterior abdominal wall), and the proximal ileum and jejunum were wrapped around the base cecum as well as ascending colon. Most of jejunum, whole of ileum, cecum, and most of ascending colon were gangrenous.

**FIGURE 2:**
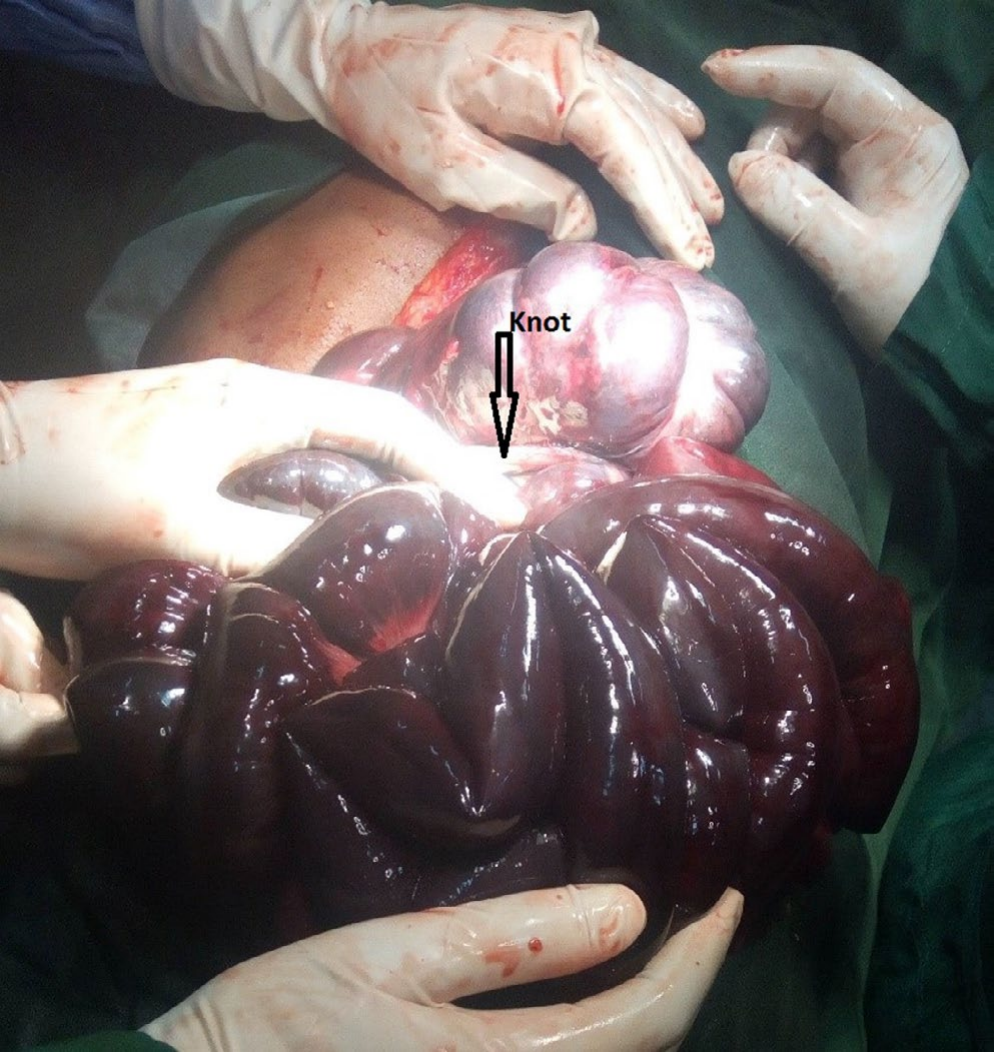
Intra‐operaive picture showing the knot

The knot was unwrapped, and the gangrenous intestine packed with warm saline for 10 to 15 minutes (Figure 2). Intestinal clamp was not used before unwrapping with the hope of saving as much intestine as possible. We were able to salvage only 40 cm of proximal jejunum, and the rest were resected. The remaining jejunum was anastomosed in two layers with transverse colon with end to side fashion. Vicryl 3‐0 was used for anastomosis. The peritoneal cavity was lavaged with 2 L of warm saline and abdomen closed. There was no drain used.

He was kept in an intensive care for 3 days but there was no need for mechanical ventilation. Later, he developed frequent diarrhea of ten to fifteen times a day starting the 4th postoperative day. This was managed with fluid replacement, antimotility drugs (loperamide), and proton pump inhibitors. Parenteral nutrition was initiated after the 10th postoperative day. The diarrhea decreased gradually, and he was discharged home after 20 days of hospital stay.

He was followed for 6 months. During this period, he lost significant weight. He also complains of weakness, crampy abdominal pain, occasional vomiting, and loose stool. Although he was being given a commercially available nutritional formula as supplement, he showed evidences of nutritional deficiencies. The patient was not checked for vitamin B12, folate, vitamins A, D, E, and K deficiencies as these tests are not routinely available here.

## 3 DISCUSSION

Intestinal knot syndromes occur when part of an intestine wraps around the base of a loop of another bowel. The most common variety is ileosigmoid knotting which occurs when an ileum wraps around the base of the sigmoid and passes beneath itself forming a knot. The earliest reported case was that of Parker in 1845, entitled, "Case of Intestinal Obstruction: Sigmoid Flexure Strangulated by the Ileum”.^3^

Since then, there are a lot of reports of iliosigmoid knotting in world literature especially from Africa including Ethiopia.^9^, ^10^

The other rarer varieties reported are ileocecal, ileoileal knots, and appendiculoileal knotting.^5^, ^11^ Operative finding in our patient was ileocecal knotting with gangrene of most of small intestine, cecum, and ascending colon. There were similar reports of ileocecal knotting by Tulsi Menon and colleagues in 2007 and by Arkaprovo Roy et al from Kolkata, India in 2011.^7^, ^12^

Intestinal obstruction by knotting is a very dangerous acute abdominal condition resulting in the development of gangrene in both loops of bowel involved in a short time. Our patient presented with a 2 days history of symptoms in a state of shock and had majority of small bowel, cecum, and ascending colon gangrenous.^3^ Diagnosis of ileocecal knotting by the usual a preoperative erect plain film of abdomen is very difficult. Certain CT features of ileocecal knotting were described by Menen T and colleagues.^7^

In this patient in addition to ileum, most of jejunum was involved in wrapping around the mobile cecum and ascending colon and all these structures were found to be gangrenous. There was only 40 cm of viable jejunum remaining, and anastomosis between jejunum and transverse colon has to be done.

The presence of a mobile cecum and ascending colon is known to predispose to midgut volvulus as well as knotting by ileum and appendix.^13^, ^14^ Thin patients with low amount of adnexal fat are more susceptible to intestinal knot syndromes although our patients’ BMI is 24.6 kg/m^2^ which is ideal.^15^

The patient developed symptoms of short bowel syndrome which was aggressively treated with fluid and electrolyte replacement as well as parenteral hyperalimentation.

Short bowel syndrome in adult is often the consequence of repeated resections for inflammatory bowel disease and occasionally for a gangrenous small bowel volvulus or mesenteric ischemia and has been reported rarely for intestinal knot syndromes.^11^, ^16^

## 4 CONCLUSION

Ileocecal knotting should be considered as a possible cause of intestinal obstruction and extensive bowel gangrene. Aggressive resuscitations, timely surgical intervention with resection of gangrenous bowel, and use of parenteral nutrition for short bowel syndrome have a good outcome.

## CONFLICT OF INTERESTS

No competing interest to disclose.

## AUTHOR CONTRIBUTIONS

The author is the surgeon who operated up on him.

## ETHICAL APPROVAL AND CONSENT TO PARTICIPATE

Ethical approval was gained from the hospital for the report.

## CONSENT TO PUBLICATION

Consent to publish was gained from the patient.

## Data Availability

All the necessary information is mentioned in text of case report.
